# Decoupled Evolution between Senders and Receivers in the Neotropical *Allobates femoralis* Frog Complex

**DOI:** 10.1371/journal.pone.0155929

**Published:** 2016-06-08

**Authors:** Mileidy Betancourth-Cundar, Albertina P. Lima, Walter Hӧdl, Adolfo Amézquita

**Affiliations:** 1 Department of Biological Sciences, Universidad de Los Andes, Bogotá, Colombia; 2 Coordenação de Pesquisas em Biodiversidade, Instituto Nacional de Pesquisas da Amazônia, Manaus, Amazonas, Brazil; 3 Department of Integrative Zoology, University of Vienna, Vienna, Austria; University of Arkansas, UNITED STATES

## Abstract

During acoustic communication, an audible message is transmitted from a sender to a receiver, often producing changes in behavior. In a system where evolutionary changes of the sender do not result in a concomitant adjustment in the receiver, communication and species recognition could fail. However, the possibility of an evolutionary decoupling between sender and receiver has rarely been studied. Frog populations in the *Allobates femoralis* cryptic species complex are known for their extensive morphological, genetic and acoustic variation. We hypothesized that geographic variation in acoustic signals of *A*. *femoralis* was correlated with geographic changes in communication through changes in male-male recognition. To test this hypothesis, we quantified male call recognition using phonotactic responses to playback experiments of advertisement calls with two, three and four notes in eight localities of the Amazonian basin. Then, we reconstructed the ancestral states of call note number in a phylogenetic framework and evaluated whether the character state of the most recent common ancestor predicted current relative responses to two, three and four notes. The probability of a phonotactic response to advertisement calls of *A*. *femoralis* males was strongly influenced by the call mid-frequency and the number of notes in most populations. Positive phonotaxis was complete for calls from each individual's population, and in some populations, it was also partial for allotopic calls; however, in two populations, individuals equally recognized calls with two, three or four notes. This evidence, in conjunction with our results from phylogenetic comparative methods, supports the hypothesis of decoupled evolution between sender and receiver in the male-male communication system of the *A*. *femoralis* complex. Thus, signal recognition appears to evolve more slowly than the calls.

## Introduction

Communication is the process of transmission of information from an individual, the sender, to another individual or individuals, the receiver(s), causing changes in the behavior of the latter [1–4]. Auditory signals are known to evolve under strong selection pressures and are important in sexual selection, speciation, species recognition, mating systems, agonistic encounters and territorial defense in many animal groups [5–7]. Advertisement calls are conspicuous and easy to record and to analyze; thus, they are widely used in taxonomy as species-specific characteristics or as indicators of reproductive isolation [8–14]. Signal variation carried out by senders in anuran communication has been studied in detail [3,15]. In contrast, the auditory perception and behavioral reactions of the receivers are much less understood [3,16,17].

A common expectation of evolutionary changes in acoustic communication is that components of communication in both senders and receivers should co-evolve gradually, as reciprocal interactions between the two can affect reproductive success [3]. Therefore, a significant change in an acoustic signal should involve a reciprocal evolutionary change in sensory processing and recognition in receivers. If changes in the signal are not accompanied by concomitant changes in the receiver sensory system, communication and species recognition may fail. Surprisingly, this aspect of acoustic signal evolution has been rarely considered. Coupled evolution between signal and signal recognition has been shown in male-female communication systems of the Túngara frog, *Engystomops pustulosus*. In this species group, a change in simple to complex calls and a corresponding change in female preference have evolved in concert multiple times in closely related lineages [18]. However, in the *E*. *pustulosus* complex, decoupled evolution among signal and signal recognition has also been observed [19–21]. Female mate choice experiments indicate that females in populations with simple calls tend to prefer complex calls (e.g., multiple chucks) rather than their own calls [19,20]. Ancestral character reconstruction analyses show that the preference for the chuck preceded the origin of the chuck, which implies that preference evolved before the trait [22], and could have been maintained by a pre-existing sensory bias [3,19,23,24]. So, within the same species complex, it is not clear how signal and signal recognition have evolved.

Because perception systems will vary depending on the type of receiver [25,26], it is important to evaluate the importance of signal-receiver coevolution in other communication systems, such as in the male-male communication system of territorial species. Male reproductive success in these species depends on characteristics other than call type, such as possession, size, and successful defense of a territory [27,28]. Although both sexes respond to the same acoustic signals, natural selection on receivers may be different: males risk losing some time and sperm, whereas the female loses much or all of her reproductive effort if she fails to recognize a conspecific's call [5,29]. This suggests that females should be more selective in their response to signal variation than males [30–32]. However, in territorial species where the recognition of conspecific intruders is key to breeding success, we expect that the sensory system of males is also is coupled to the signal.

### Ecology and behavior of the *Allobates femoralis* Complex

*Allobates femoralis* (Anura: Aromobatidae) is a complex of cryptic species widely distributed in the Amazonian basin [33–36] (S1 Fig). Increased acoustic and phenotypic variation as well as substantial genetic divergence among populations has been observed [34]. Communication studies of this species [34,37–41] suggest that it is an excellent model to evaluate mechanisms of perception and recognition of acoustic signals by receivers because males use advertisement calls to attract females and to announce their readiness to defend their territory from conspecific male intruders; moreover, males respond very well to playback experiments [37,38]. As in other poison frogs [27,42–45], reproductive success of *A*. *femoralis* males is strongly related to ownership, size and successful defense of a territory [27,28]; thus, a male who fails to detect conspecific intruders not only risks losing its territory, but also risks losing mating opportunities. Likewise, *A*. *femoralis* males respond to stimuli with spectral characteristics (frequency) that are beyond the range of natural variation [46]. The number of notes in calls of the *A*. *femoralis* complex varies geographically, but it is unknown whether geographical variation in the signals also involves changes in signal recognition. The geographic variation of calls in this system allows us to assess the hypothesis that signals and signal recognition evolve together. This assumption should be evaluated because it is often the starting point of many taxonomic, communication, and speciation studies.

**Fig 1:**
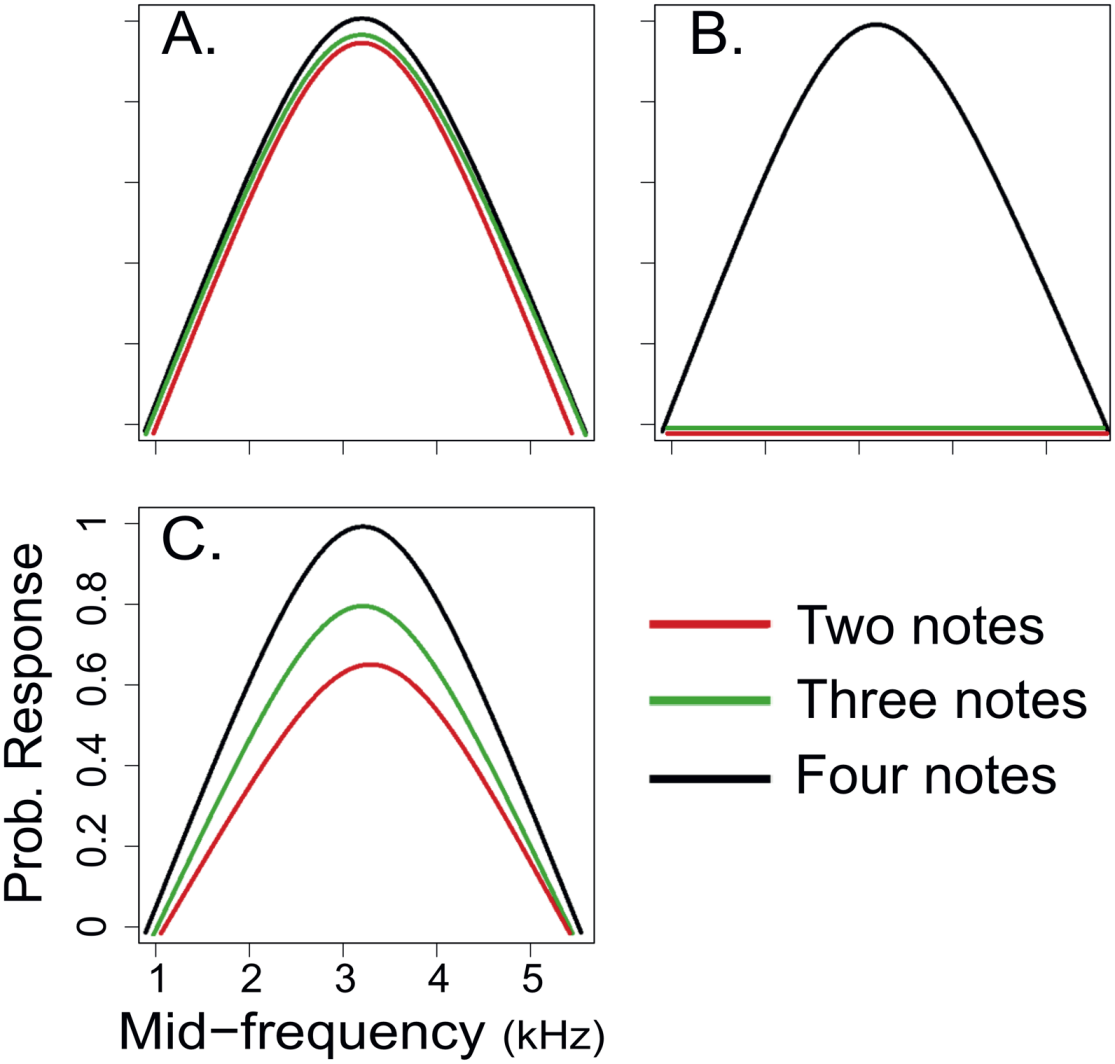
Probability of phonotactic response by males of *A*. *femoralis* to synthetic advertisement calls. Calls varied in frequency and number of notes per call. We test a population that has four-note calls. (A) Null scenario, where the population responds to all variations in the number of notes. (B) Matched-spaces hypothesis, where the population only recognizes the variant number of notes that corresponds to own advertisement calls. (C) Intermediate scenario, where the population is most likely to recognize their own calls but also partially recognizes other call variants. Scenarios A and C suggest a process of decoupled evolution for signal and signal recognition. Colors represent variations in number of notes: red, two notes; green, three notes; and black, four notes.

The central aim of this study was to test the hypothesis that there is decoupled evolution between sender and receiver in the male-male communication system of the *A*. *femoralis* complex. We determined whether geographical variation in advertisement calls produces synchronous changes in signal recognition using phonotactic response and phylogenetic comparative methods. Phonotactic responses are an established method to test acoustic signal recognition and preferences; it has been used in previous studies of the male-male communication system of *A*. *femoralis* complex as well as in other territorial species [37,38,41,47]. We propose three scenarios (Fig 1). First, our null hypothesis predicts that the probability of call recognition by males does not vary with number of notes in a call (two, three and four notes). This result would indicate completely decoupled evolution between signal and receiver. Second, if space signal recognition is perfectly coupled to the space signal, i.e. the "matched-spaces hypothesis" [46], we predict that males should recognize only the calls of other males from the same population. Finally, we proposed an intermediate scenario, which includes a range of possibilities, such as total recognition of males’ own calls but partial recognition of calls from other populations. This result would also suggest a scenario of decoupled evolution.

### Phylogenetic analysis of call evolution

**Fig 2:**
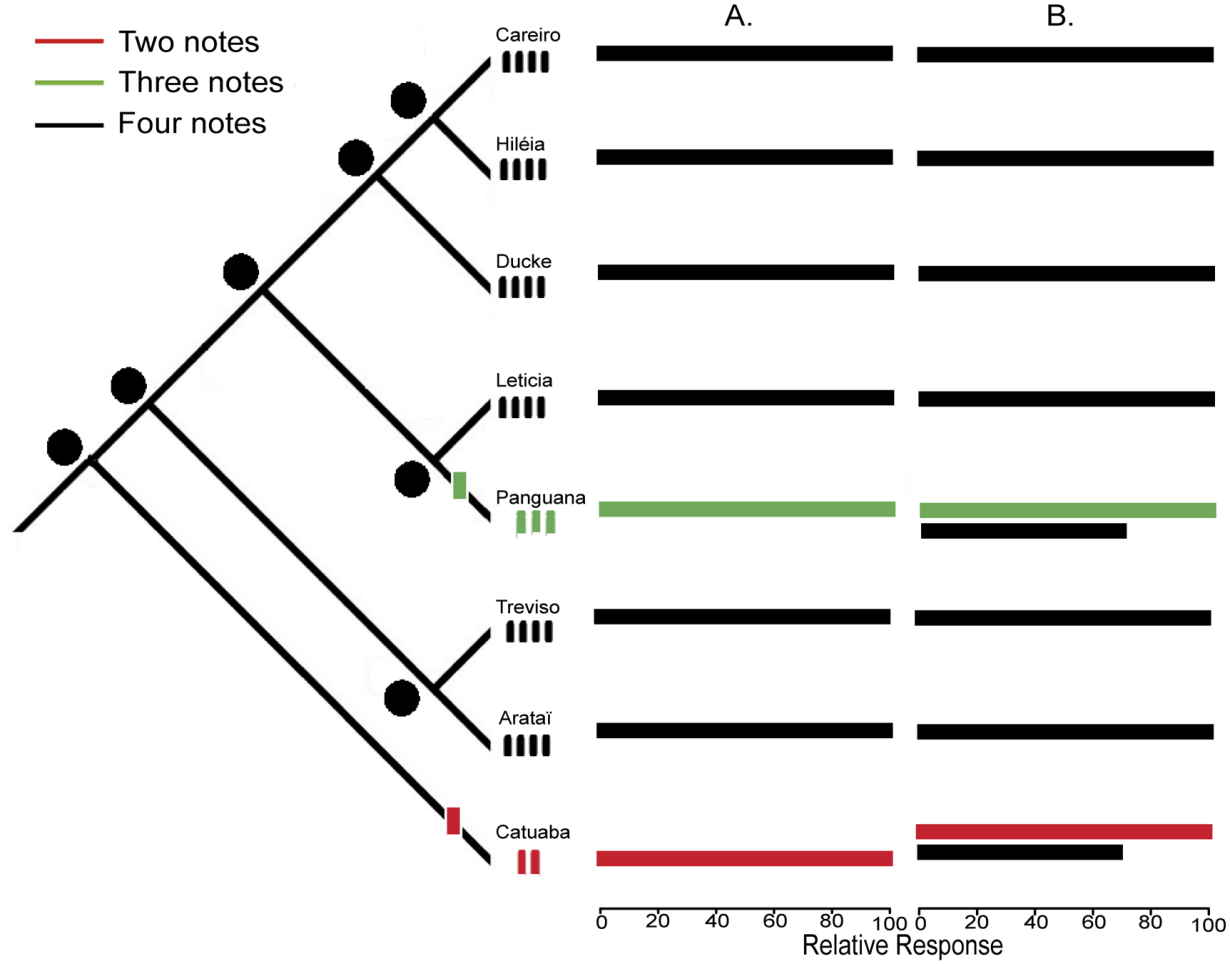
Ancestral state reconstruction and relative response to three variations in the number of notes. Ancestral states of the number of notes (left) and the relative response to variations in the number of notes (right) according to coupled (A) and decoupled (B) evolutionary scenarios. Phylogenetic relationships are based on previous work [34,35]. We display one of the most parsimonious scenarios including two changes in the number of notes (two and three notes) and an ancestor of four notes for all nodes, as this state is present in most populations (79.5% of populations). Colors indicate the number of notes per call and relative response to two notes (red), three notes (green) and four notes (black) for each population.

In our phylogenetic analyses, we propose two scenarios: 1) signal and signal recognition evolve at a similar rate. In this case, would expect each lineage to recognize only the variation in the number of notes of the same population; e.g., if a lineage calls with three notes, it will only recognize its own three-note calls (Fig 2A). 2) Signal and signal recognition change at a different rate; in this case, we would expect that the character state of the most recent common ancestor of this group predicts the contemporary response to calls with two, three and four notes. A clade that fits this scenario would most likely recognize its own calls, but would also partially recognize the variation in the number notes present in its ancient relatives. For example, if a clade with two-note calls was derived from an ancestor with four-note calls, then the descendant clade likely still recognizes four-note calls. This scenario indicates that the signal is changing faster than signal recognition (Fig 2B), suggesting decoupled evolution between these two traits. To evaluate these hypotheses, we first quantified male recognition of calls with two, three, and four notes using playback experiments. Second, we reconstructed the ancestral states of call note number in a phylogenetic framework, and third, we evaluated whether the predicted number of notes in the call of the most recent common ancestor predicts the relative response to calls with two, three and four notes.

## Materials and Methods

### Ethics statement

All applicable international, national, and/or institutional guidelines for the care and use of animals were followed. All procedures performed in studies involving animals were in accordance with the ethical standards of the Universidad de Los Andes and the Colombian Government; Procedures for recordings, capture, and handling of live animals in the field were approved by CORPOAMAZONIA under research permits 1128 of November 8–2002 in Colombia, 004/03 of IBAMA/RAN in Brazil and 078–2003 of INRENA in Peru.

### Study area

We collected information on the number of notes per call from 14 localities distributed across the Amazon basin (S1 Table and S1 Fig) [34,35] to estimate ancestral states. Advertisement calls show variation ranging from calls composed by a single note (Ecuador) [48] to calls composed of four notes [34]. This variation is correlated with geographic distance and genetic variation [34] or with geographic barriers [36]. To assess whether there is differential recognition of *A*. *femoralis* to calls with different numbers of notes, we carried out playback experiments at eight localities throughout the Amazon basin (Table 1 and S1 Fig) [34]. Advertisement calls consist of two notes in the Catuaba population (*Allobates hodli*), three notes in Panguana, and four notes in other populations [34]. We consider *A*. *hodli* as part of the *A*. *femoralis* complex because phylogenetic studies reveal that these populations are nested within *A*. *femoralis* [35,49].

**Table 1:** Values of significance (P-value) and explained variance obtained for the eight populations of *A*. *femoralis* using Generalized Additive Models—GAM.

Population	Country	Coordinates(Lat, Long)	Number of notes	Mean peak frequency (kHz)	N	P-value		Explained variance (%)
						Number of notes	Mid-Frequency	
Careiro	Brazil	(-3.3547, -59.8605)	4	3.12	72	0.012*	0.0023*	50.7
Leticia	Colombia	(-4.1233, -69.9491)	4	3.08	67	0.043*	0.0131*	60.0
Hiléia	Brazil	(-3.1977, -60.4425)	4	3.10	89	0.013*	0.0065*	32.8
Ducke	Brazil	(-2.9333, -59.9744)	4	2.87	73	0.025*	0.2690	57.7
Panguana	Peru	(-9.6137, -74.9355)	3	3.07	72	0.010*	0.2030	60.2
Catuaba	Brazil	(-10.0742, -67.6249)	2	3.34	72	0.452	0.0146*	34.4
Treviso	Brazil	(-3.1491, -54.8403)	4	3.33	80	0.052	0.0003*	32.9
Arataï	French Guiana	(3.9907, -52.5901)	4	3.44	68	0.010*	0.0004*	44.5

The predictors were number of notes and mid-frequency (kHz). We indicate the number of playback experiments (N) conducted in each population.

* values significant with respect to a p-value of 0.05.

### Behavioral experiments

To carry out the experiments we strictly followed previously published methods [37,38] described in detail in S1 Text. Briefly, we carried out the following steps. 1). We located the territory of a male *A*. *femoralis* and recorded and analyzed auditory signals. We used Raven Pro 1.5 [50] to measure call parameters following previously protocols [51]. We measured the spectral (peak, low and high frequency) and temporal (call duration, inter-call interval, note duration, inter-note intervals) features of each call in order to prepare the synthetic stimuli. 2). We prepared the synthetic calls by systematically changing the number of notes and frequency based on parameters of the Leticia population [37]. Additionally, we synthesized a “control” call according to the average temporal and spectral parameters of each population. All stimuli were synthesized with SoundEdit 2.0.3 software [52]. 3). We conducted 593 playback experiments in eight localities of the Amazonian basin (S1 Fig). These experiments were conducted following protocols published elsewhere [37,38,53]. All tests were performed between 05:30 and 11:00 hours and 15:30 and 18:25 hours under rainless conditions, when the calling activity of *A*. *femoralis* was high [27,54]. We avoided pseudoreplication by using a different signal in every trial [37,47]. Likewise, a maximum of two experiments separated by a period of no calling activity (one night or one afternoon) were performed with a single male. Additionally, we never tested two calls of similar frequency on the same individual. 4). Signal recognition was quantified by a binary phonotactic response. If the tested male crossed a perimeter of 30 cm around the loudspeaker within the 10 bouts of 10 calls of the stimulus, we stopped the experiment and declared a positive phonotactic response. If a male did not approach the speaker, we used a second stimulus (“control”). If the male recognized its stimulus, we assumed that it failed to recognize the first stimulus and we quantify it as negative response. Any subject that failed to respond to both the experimental and the control stimuli was excluded from statistical analyses.

### Statistical analysis

We used a generalized additive model (GAM) [55] to model the species’ recognition space because these models use multiple predictors, incorporate nonparametric functions that are better adapted to nonlinear relationships among data, and can include variables that do not fit to a normal distribution [55,56]. This fits our data because the analyzed response variable was binary (1 or 0): The males either recognized the stimulus and approached the speaker (1) or failed to recognize it (0). Our predictors were one spectral (Mid-Frequency, kHz) and one temporal (number of notes) call parameter. We analyzed the data using the 'mgcv' package in R (Mixed GAM Computation Vehicle with GCV/AIC/REML smoothness estimation) [57]., We used a Bonferroni correction [58] with the formula *p* = *α*/*k* where *α* = 0.05 and *k* is a the number of population comparisons (8), for a p-value of 0.00625.

### Ancestral state reconstruction

The aim of this study was not to determine phylogenetic relationships among different populations of the *A*. *femoralis* complex. However, there is no robust phylogeny for this species complex. Thus, we conducted a phylogenetic analysis using 91 sequences of two mitochondrial genes, cytochrome b (Cyt b) with 357-bp and 16S with 508-bp. The sequences were obtained from previous studies [34,35] and GenBank (S1 Table), and we used *Allobates zaparo* as an outgroup. We aligned the sequences using MUSCLE [59] implemented in Geneious 7.1.2 [60]; the resulting alignments were visually corrected to resolve gap placements and were subsequently concatenated. In order to estimate phylogenetic relationships within the *A*. *femoralis* complex, we employed two phylogenetic approaches, Maximum likelihood (ML) [61] and Bayesian analysis [62]. The ML analysis was conducted in MEGA 6.0. [63] with 2000 bootstrap replicates to assess nodal support and under the T92+G (Tamura 3-parameters) model which was selected as the best-fit to the data according to the Bayesian Information Criterion implemented in jModelTest 2.1.3 [64]. The Bayesian phylogeny was inferred using Beast 1.7 [65] under the priors Yule-process and HKY+G model. This model was considered the most similar to T92+G given the constraints of the software. The chains were run for 30,000,000 generations, sampling every 1000 and discarding the first 7500 (25%) trees as burn-in. Chain convergence was examined using Tracer [66]. We then randomly selected a single individual per population from the existing dataset. With these data, we conducted another phylogenetic analysis using the same approaches and parameters mentioned above (except for a 50% burn-in). The selection of individuals was trivial, because most individuals formed reciprocally monophyletic groups except for two individuals from Panguana, Peru.

Because the phylogenetic relationships using both ML and Bayesian approaches were similar, we estimated ancestral character states for the number of notes under the maximum clade credibility tree. This character was tested as a discrete-nominal with four states: one, two, three and four notes. We used the algorithm "rerooting Method" in the Phytools package [67] in R. Likelihood reconstruction methods find ancestral states that maximize the probability that the observed states would evolve under a stochastic model of evolution [68,69]. We used the model MK1 (Markov k-state 1 parameter model) [70] or "ER" (in the language of R). Any particular change (from state 1 to 2 or state 1 to 4, for example) is equally probable. To consider the effect of phylogenetic uncertainty on inferences of trait evolution, we estimated ancestral states for number of notes using ML across the 15,000 post-burn-in trees, and summarized patterns of change across all these trees in Mesquite [71].

### Quantification of relative response

To compare the relative response to each stimulus (calls of two-notes versus three-notes or four-notes), we calculated the area under the curve (AUC) of the response probability. This was estimated by the GAM models for each of the stimuli. The AUC was calculated with the MESS package (Miscellaneous esoteric statistical scripts) [72] in R. The AUC or relative response of each stimulus was re-calculated as a proportion, with respect to each AUC value, that corresponds to the call recognition of the same population. This variable was used in the analysis to assess whether the character state of the most recent common ancestor predicted the relative response to calls with two, three and four notes. We mapped the variation in the number of notes on the phylogeny, and presented it graphically, integrating information from the eight populations.

## Results

**Fig 3:**
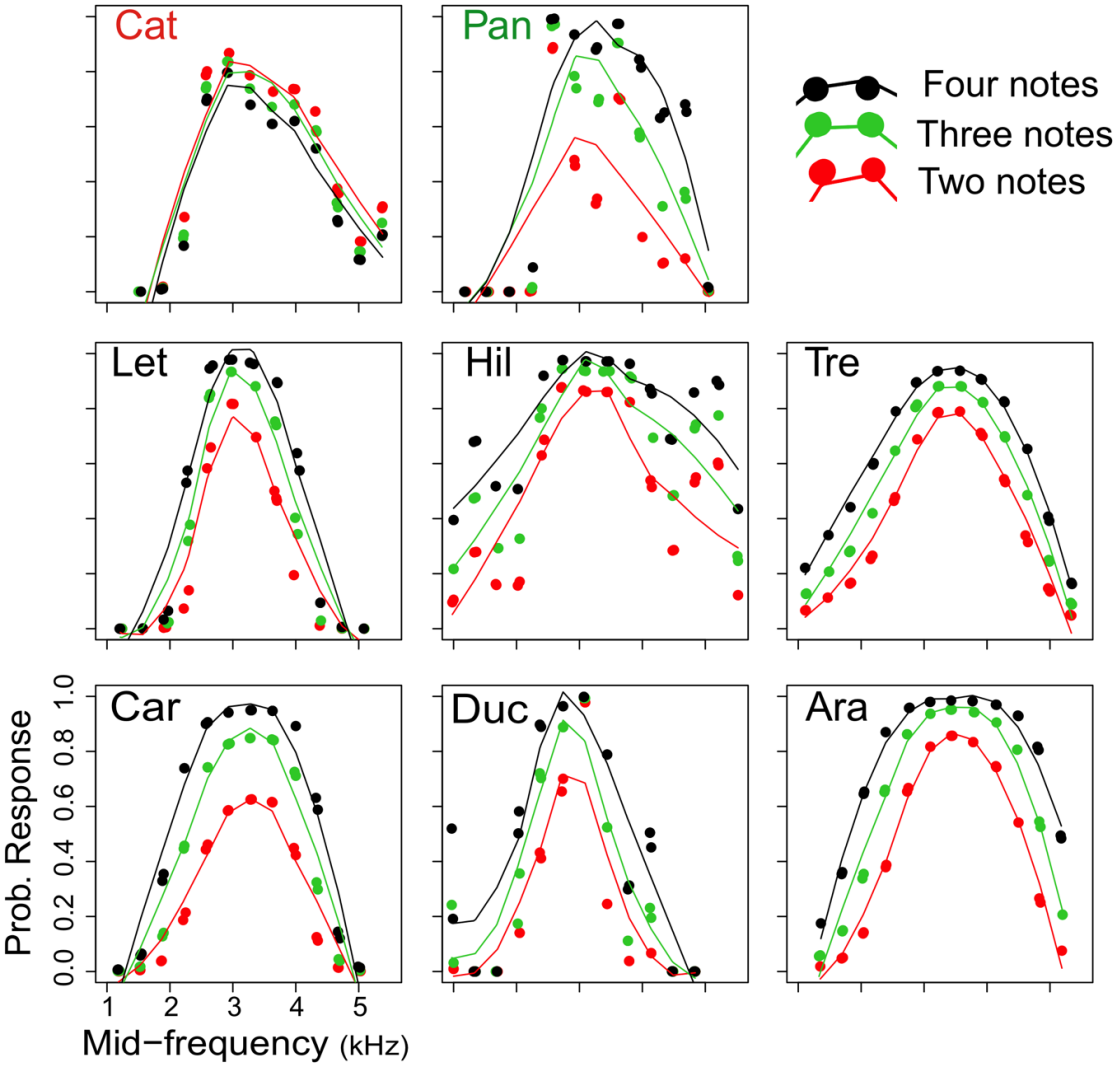
Probability of phonotactic response of *A*. *femoralis* males to synthetic advertisement calls. Calls varied in frequency and number of notes. Colors indicate variations in the number of notes: two notes in red, three in green and four notes in black. Populations vary in the number of notes per call: Catuaba (Cat) has two notes (*A*. *hodli*). Panguana (Pan) three notes, and Leticia (Let), Hiléia (Hil), Treviso (Tre), Careiro (Car), Ducke (Duc) and Arataï (Ara) have four notes. Probability of phonotactic response was predicted by the GAM models.

The probability of male *A*. *femoralis* response to calls was mainly explained by two variables, number of notes and mid-frequency. The percent variance explained by these two predictors ranged from 32.8% for the population from Hiléia to 60% for Leticia (Table 1). The maximum probability of male response towards variations in the number of notes coincided with the average frequency of the advertisement call of each study population. These values ranged from 2.87 kHz (Ducke) and 3.44 kHz (Arataï) (Fig 3 and Table 1).

These *A*. *femoralis* males recognized all variations in the number of notes; however, in some cases the probability of signal recognition varied according to the number of notes in each call (Fig 3). Treviso and Catuaba (*A*. *hodli*) populations did not show any statistically significant differences in the probability of recognition of the three note variants (P = 0.052 and 0.452, respectively) (Table 1). Populations of males that called with four notes showed a similar pattern, where the highest probability of recognition corresponded to four-note calls, then three-note and lastly two-note calls (Table 1 and Fig 3). The population calling with three notes (Panguana) presented an interesting case. Significant differences in the signal recognition of the three variants in the number of notes (P = 0.010) were revealed, but we found that the greatest probability of recognition was for four-note calls (Fig 3). After Bonferroni correction (P = 0.00625), there were no significant differences in recognition of two, three and four notes for all populations tested (Table 1).

**Fig 4:**
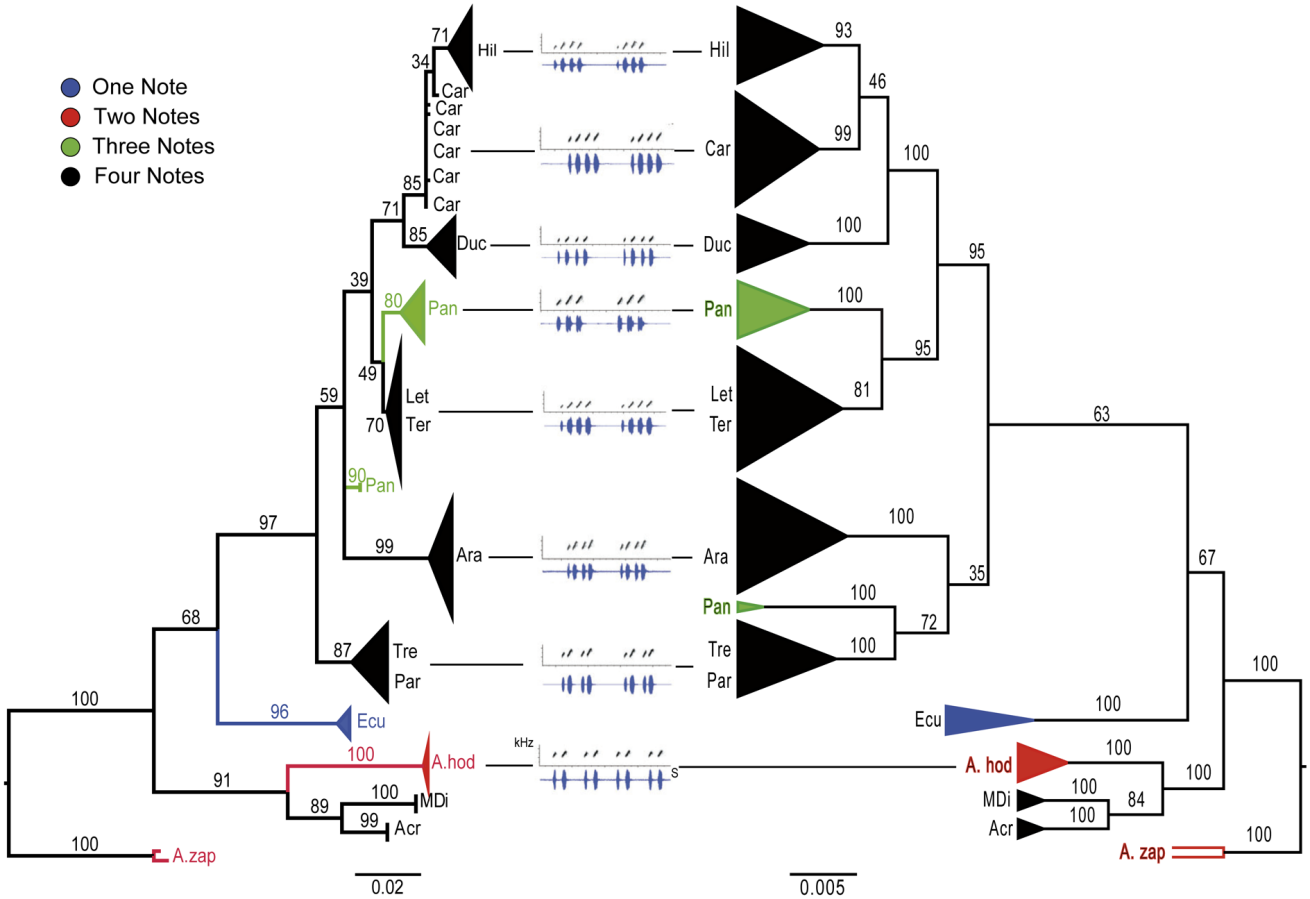
Phylogenetic hypotheses conducted under maximum likelihood (left) and Bayesian approaches (right). Phylogenies were estimated using 91 sequences of mitochondrial genes Cyt b (357 bp) and 16S (509 bp). The values at the internal nodes indicate bootstrap support and posterior probability, respectively. The current state of the number of notes in advertisement calls is denoted by clade color; oscillograms (blue) and sonograms (gray) between phylogenies show 2-s recordings of at least two advertisement calls for each study population (Modified from: Amézquita et al, 2009). Colors indicate the composition of the number of notes: blue indicates one note, red, two notes, green, three notes, and black, four notes. The names on the tips of the branches refer to *A*. *zaparo* (A. zap), *A*. *hodli* (A. hod). Acre (Acr), Madre de Dios (MDi), Ecuador (Ecu), Pará (Par), Treviso (Tre), Arataï (Ara), Panguana (Pan), Type locality (Ter), Leticia (Let) Ducke (Duc) Careiro (Car) and Hiléia (Hil) correspond to populations of *A*. *femoralis*.

Our phylogenetic analyses based on mitochondrial genes (Cytb and 16s) suggested high genetic structure between populations since most individuals formed reciprocally monophyletic groups (Fig 4). Our results largely confirm previously published studies [35] where *A*. *zaparo* was found to be the sister group of all *A*. *femoralis* populations; this clade was sister to *A*. *hodli* and the *A*. *femoralis* populations from Acre and Madre de Dios (Fig 4). Phylogenetic relationships among populations of Treviso and Arataï are ambiguous, because the support for these nodes in ML and Bayesian approaches was low (bootstrap support = 59 and posterior probability = 35), in addition the phylogenetic relationships do not match between the two approaches (Fig 4).

**Fig 5:**
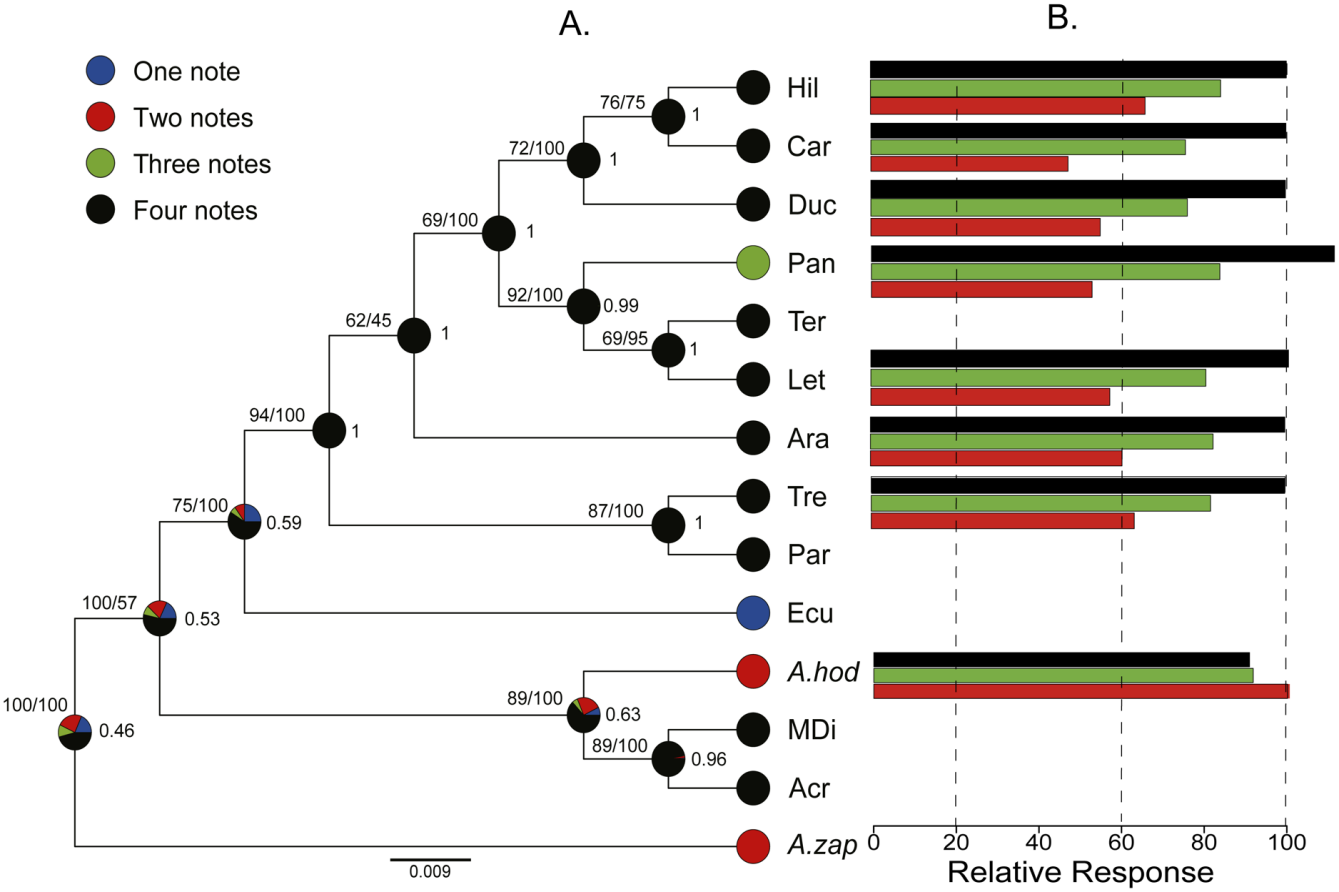
Ancestral character reconstruction for number of notes (A) and relative response for this character (B). Relative response to each change in the number of notes was calculated as a ratio to the area under the curve (AUC). Estimation of ancestral states was carried out with the maximum credibility tree obtained from the Bayesian analysis under the ML criterion and the MK1 model. The values associated in front of each node indicate the likelihood that the males had called with four notes and the values above each node show the bootstrap support for the posterior probability, respectively. The area of each color in the circles at the nodes indicates the relative support for different ancestral states of call note number. The colors refer to the number of notes in the advertisement calls of each population (on the tree) and the relative response. Blue indicates one note, two notes are in red, three notes in green, and four notes in black. The names on the tips of the branches refer to *A*. *zaparo* (A. zap), *A*. *hodli* (A. hod). Acre (Acr), Madre de Dios (MDi), Ecuador (Ecu), Pará (Par), Treviso (Tre), Arataï (Ara), Panguana (Pan), Type locality (Ter), Leticia (Let) Ducke (Duc) Careiro (Car) and Hiléia (Hil) correspond to populations of *A*. *femoralis*.

The ancestral state reconstruction of note number at the basal nodes is ambiguous, so we were unable to infer which call note variant was present in ancestral lineages. The most likely ancestral state of the number of notes in the call of the ancestor of the *A*. *femoralis* complex is four, with a 53% probability (Fig 5A). The remaining 47% probability is distributed among one, two, and three notes. In more recent lineages, reconstruction becomes more consistent with an ancestor of four notes (probability = 100%). Thus, the only evolutionary change in call note number that can be inferred is that of the Panguana population, which changed from four to three notes (Fig 5A).

The analysis that incorporated phylogenetic uncertainty showed similar results (S2 Fig). The ancestral state for the number of notes in the basal nodes is ambiguous and the probability for a state of four notes in more derived nodes is greater (S2 Fig). Thus, we emphasize that uncertainty in phylogenetic reconstruction does not affect our interpretation of results because our inferences are not based on a single tree, but rather on analyses conducted across a large sample of plausible trees sampled from the posterior distribution in our Bayesian analysis. The summary of changes in the number of notes on all the trees indicated seven possible changes (S2 Table). The only change we can infer with certainty is from four to three notes for the Panguana population. Finally, the results in the recognition of two, three and four notes suggest that all populations, regardless of the number of notes in their advertisement calls, recognize the three variants of the call. The higher relative response for all populations was for the calls of their own population, except for the population with three-note calls, which demonstrates a greater recognition to the stimulus of four notes (Figs 3 and 5B).

## Discussion

The probability of male response to synthetic calls in the *A*. *femoralis* complex was strongly influenced by two characteristics: a spectral parameter, the mid-frequency call of each population, and a temporal parameter, the number of notes. As in previous studies [37,38], the probability of the response curve showed a steeper decline at frequency values below and above the population average of each population.

Our results allow us to reject the hypothesis of matched-spaces [46]. Therefore, our data do not support a process of coupled evolution between the number of notes and signal recognition. However, both of the remaining scenarios (our null hypothesis or intermediate scenarios) were supported by our data, depending on the study population. In Catuaba (*A*. *hodli*) and Treviso (*A*. *femoralis*) populations, no differences were found in the recognition of two, three and four notes. This evidence supports the null hypothesis, in which the probability of recognition by males is equal for the three note variants. In populations where males call with four notes, we found differences in the probability of recognition of each note variant, i.e. *A*. *femoralis* males were more likely to recognize their own advertisement calls, but they also recognize two- and three-note variants. This evidence supports the hypothesis of our intermediate scenarios. After Bonferroni correction, all populations were compatible with the null hypothesis, rather than the intermediate scenario. This correction allowed us to determine which decoupled evolution scenario (null hypothesis or intermediate scenarios) fit our data, but it never contradicted our central hypothesis.

The two ancestral character reconstruction analyses showed ambiguous results for the basal nodes. Consequently, these estimates did not allow us to infer the ancestral state of number of notes. However, in recent lineages, there was higher support for an ancestral state of four notes. This character state is found in most populations of the *A*. *femoralis* complex. In 44 existing populations, 79.5% call with four notes (35 populations) [35]. The uncertainty in the reconstruction of ancestral call note states could be associated with the greatest variation in the number of notes present in basal lineages (one, two and four notes).

Our phylogenetic and behavioral evidence allows us to reject a scenario of coupled evolution in signal and signal recognition of *A*. *femoralis*. Our results fully support the decoupled evolution scenario, where recognition evolves slower than the signal. We cannot determine whether the phylogenetic position of a clade predicts the recognition of variations in the number of notes, as the estimation of ancestral states was ambiguous for the basal nodes and all populations recognize the three note variants. However, these basal populations (*A*. *hodli*, Arataï and/or Treviso) also support our hypothesis, because they recognize the three-note call, despite the absence of an ancestral history of this state.

A mechanism of decoupled evolution between sender and receiver was previously suggested to exist in male-female communication systems [19–21], where the breeding success of males depends exclusively on the ability to produce complex calls. Our study was different because we evaluated a system of male-male communication, and males have been suggested to have a less selective sensory system than females [5,29]. Second, the breeding success of males is determined by size (0.25 to 26 m^2^) and successful defense of their territory [27]. It is likely better than not for males of the *A*. *femoralis* complex to approach a stimulus that sounds like a conspecific, or else they may experience higher costs than energy expense such as losing their territory due to failure of signal recognition. This trade-off may have helped maintain a flexible sensory system, given the large variation in the number of notes of *A*. *femoralis* populations (one to six notes) [73]. Considering that males use advertisement calls to attract conspecific females and defend their territory from conspecific males [27,74], it would be interesting to evaluate the role that females may have played in communication system evolution. However, in preliminary trials, females do not respond to playback recordings (Pers. Com. A. Amézquita) so females may not be evaluating the male call as much as their territory. If this is true, females may act as an indirect selection pressure on male-male communication, favoring the pattern of broad signal recognition in males.

The communication system of *A*. *femoralis* males probably does not reflect a generalization for territorial species unless other species share their same reproductive strategies. It could be a special case. For example, in a population where *A*. *femoralis* coexists in the acoustic environment with the highest number of species (10 species in Panguana) [38], it has been shown that males are capable of recognizing a greater range of spectral parameters that are beyond the natural range of the signal [46].

An important observation from our study is that the population calling with three notes (Panguana) had a higher probability of recognition for four-note advertisement calls than its own calls. This could be related to the phylogenetic position of this clade within the *A*. *femoralis* complex because their divergence is relatively recent and their ancestor likely called with four notes. This suggests that the number of notes evolved faster than signal recognition. Although populations have diverged in temporal characteristics, it is likely that the sensory system of the receiver still recognizes the calls of their ancestors. However, we have no population replicates to confirm this hypothesis. Likewise, it has been suggested that perfect coupling between sender and receiver does not allow a communication system to evolve, so signal and signal recognition should not be evolutionarily stable systems [23].

As *A*. *femoralis* is a highly territorial species, a plausible interpretation to explain the change in the advertisement calls to three notes for this population might be related to occurrence of character displacement in interspecific interactions mediated by calls to defend resources and territories. In Panguana, *A*. *femoralis* coexists with ten species. In this acoustically dense environment, at least one species, *Ameerega petersi*, has a call that overlaps in spectral features (peak frequency and frequency range) and pattern of call with that of *A*. *femoralis* [38,46]. This overlap could have promoted the shift to three-note calls in this *A*. *femoralis* population. Character displacement in both temporal and spectral variables, which decreases acoustic interference with other species, has been previously documented in *A*. *femoralis* [38,41], in other dendrobatids [75], and in other species of amphibians [76–78].

Advertisement calls have been widely used in a taxonomic context as unique characteristics of species, as they are closely related to mechanisms of reproductive isolation and intersexual communication [9,8,79]. Our study suggests that, at least in the system of male-male communication, temporal variation in a trait call does not represent an isolation mechanism for intra-sexual communication, as males recognize more than one call note variant. However, to predict reproductive isolation it is necessary to assess female recognition of different call stimuli. However, *A*. *femoralis* females do not exhibit territorial behaviors and mating preference appears to be associated with ownership, size and successful defense of a territory; in addition, they are likely to mate with any spatially proximate male. [27,28]. Acoustic signals may be under different selection pressures; therefore, the use of only calls as a tool to define or delimit species in systematics and taxonomy should be implemented with care.

## Supporting Information

S1 Fig Distribution of study populations of *Allobates femoralis* throughout the Amazon basin. (PDF)

S2 Fig Ancestral character reconstruction of note number of the *A*. *femoralis* complex. (PDF)

S1 Table Information of DNA sequences used in the phylogenetic analysis. (XLSX)

S2 Table Summary of changes in call note number. (PDF)

S1 Text Detailed description of the study methods. (PDF)
