# Supplementary material for: Decoupled Evolution between Senders and Receivers in the Neotropical Allobates femoralis Frog Complex
Source: PLoS One. 2016 Jun 8;11(6):e0155929. doi: 10.1371/journal.pone.0155929 (PMC4898772; doi:10.1371/journal.pone.0155929)

S1 Figure. Distribution of study populations of *Allobates femoralis* throughout the Amazon basin. Colors indicate variations in the number of notes in advertisement calls: one note in blue, two in red, three in green and four notes in black. Circles represent the *Allobates femoralis* populations, red circles are *A. hodli* populations and rhombus represent *A. zaparo* populations. We conducted experiments playback in Leticia, Catuaba (*A. hodli*), Panguana, Hiléia, Treviso, Careiro, Ducke and Arataï populations.

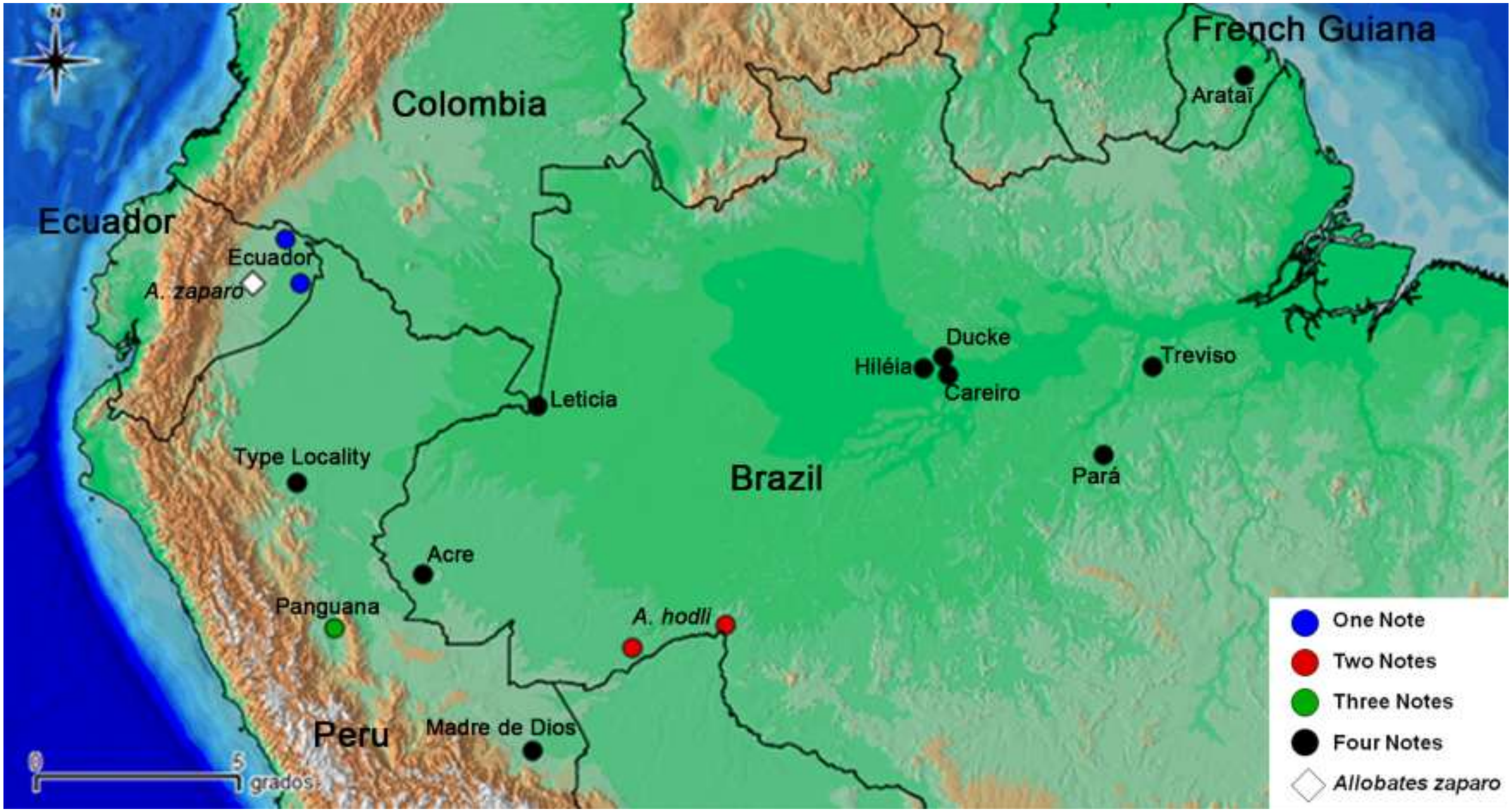

Supplement: S1 Fig — (PDF) [file pone.0155929.s001.pdf]
