# Supplementary material for: Decoupled Evolution between Senders and Receivers in the Neotropical Allobates femoralis Frog Complex
Source: PLoS One. 2016 Jun 8;11(6):e0155929. doi: 10.1371/journal.pone.0155929 (PMC4898772; doi:10.1371/journal.pone.0155929)
Supplement: S2 Fig — (PDF) [file pone.0155929.s002.pdf]

S2 Figure. Ancestral character reconstruction of advertisement call note number of the *A. femoralis* complex. Estimation of ancestral states was carried out under the ML criterion and the MK1 model. We incorporate phylogenetic uncertainty with 15,000 trees sampled from the posterior distribution obtained in the Bayesian analysis. We present our data on the maximum credibility tree. The values associated with each node indicate the posterior probability. The area of each color in the circles at the nodes indicates the relative support for different ancestral states; colors refer to the number of notes in the advertisement calls of each population (on the tree) and/or the relative response. Blue indicates one note, two notes are in red, three notes in green and four notes in black. White indicates the proportion of trees in which the node was absent and gray indicates the relative likelihood of inconsistency in the reconstruction of the ancestral state for a given node according to all the trees. The names on the tips of the branches refer to *Allobates zaparo* (A. zap), *A. hodli* (A. hod). Acre (Acr), Madre de Dios (MDi), Ecuador (Ecu), Pará (Par), Treviso (Tre), Arataï (Ara), Panguana (Pan), Type locality (Ter), Leticia (Let) Ducke (Duc) Careiro (Car) and Hiléia (Hil) correspond to populations of *A. femoralis*.

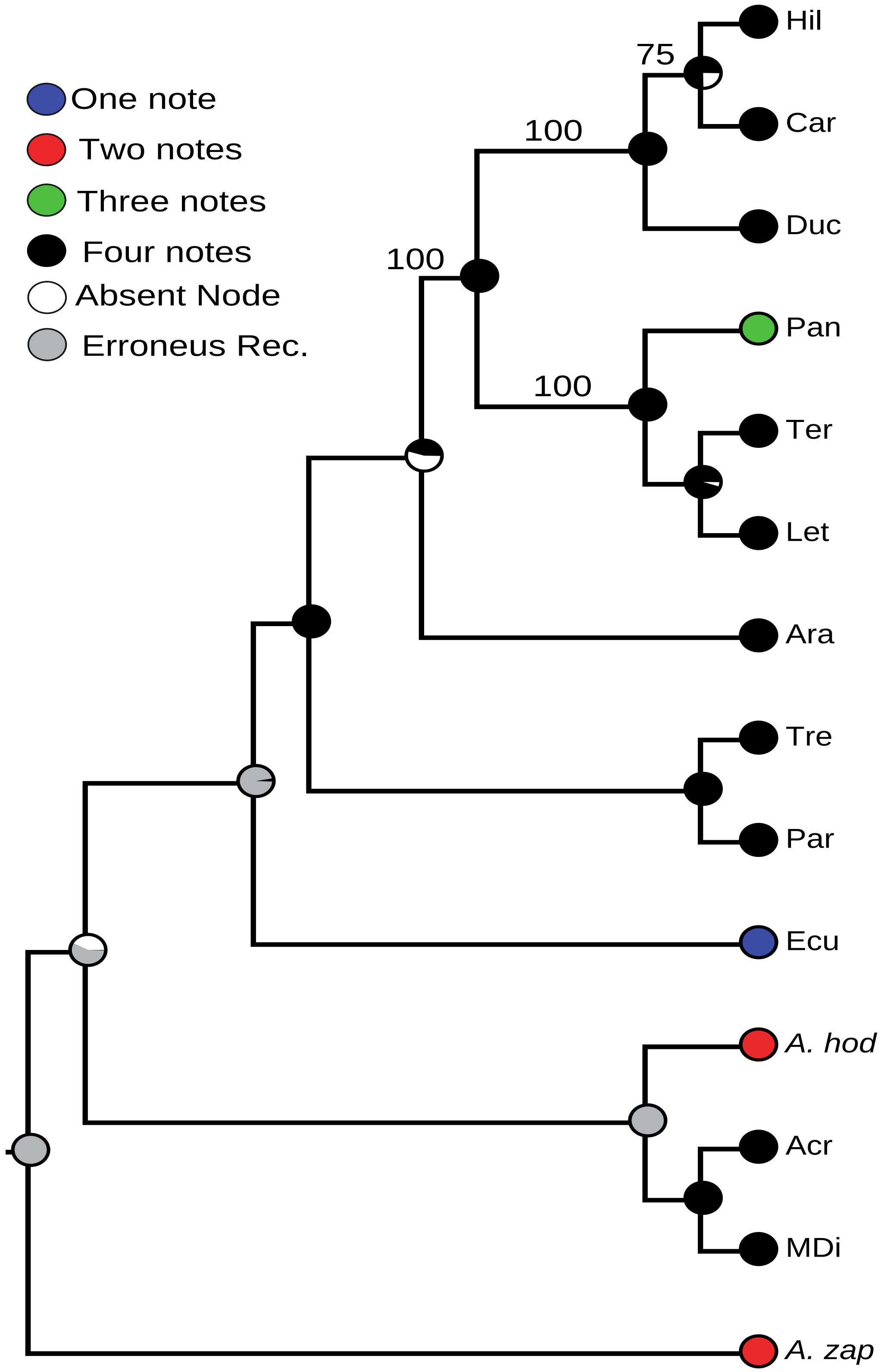
One note
Two notes
Three notes
Four notes
Absent Node
Erroneus Rec.
75
100
100
100
Hil
Car
Duc
Pan
Ter
Let
Ara
Tre
Par
Ecu
A. hod
Acr
MDi
A. zap
