# Supplementary material for: Decoupled Evolution between Senders and Receivers in the Neotropical Allobates femoralis Frog Complex
Source: PLoS One. 2016 Jun 8;11(6):e0155929. doi: 10.1371/journal.pone.0155929 (PMC4898772; doi:10.1371/journal.pone.0155929)
Supplement: S2 Table — (PDF) [file pone.0155929.s004.pdf]

S2 Table. Summary of changes in call note number over 15,000 trees sampled from the posterior distribution obtained in the Bayesian analysis. The summary of changes in the number of notes on all the trees indicated seven possible changes. We indicate the type of change, the average, minimum and maximum number of changes and the percentage of trees in which the change occurred.

Change	Min	Max	Average	% of trees
1 to 2	0	1	0,028	3
1 to 4	0	1	0,235	23
2 to 1	0	1	0,415	41
2 to 4	0	2	1,206	78
4 to 1	0	1	0,557	55
4 to 2	0	2	0,559	34
4 to 3	1	1	1,000	100
