# Supplementary material for: Decoupled Evolution between Senders and Receivers in the Neotropical Allobates femoralis Frog Complex
Source: PLoS One. 2016 Jun 8;11(6):e0155929. doi: 10.1371/journal.pone.0155929 (PMC4898772; doi:10.1371/journal.pone.0155929)
Supplement: S1 Text — (PDF) [file pone.0155929.s005.pdf]

S1 Text. Detailed description of the study methods.

Recording and analysis of auditory signals

To carry out the experiments at each locality, we first located the territory of a male *A. femoralis* and recorded their advertisement calls without capturing them to minimize our impact on frogs' motivational state and acoustic environment. To characterize signals, we tape recorded (Sony WM D6C or Marantz PMD660 recorder) between 3 and 34 spontaneous and consecutive calls with a microphone (AKG D-190-E o Shure BG4.1) positioned at a distance of 0.5 m to 1.5 m in front of each male. Second, we measured the spectral (peak, low and high frequency) and temporal features (call duration, inter-call interval, note duration, inter-note intervals) of each call to prepare the synthetic stimuli necessary for playback experiments. Recordings were digitized at 22 kHz and spectral parameters of the calls were analyzed, calculating power spectra (Window: Blackman, DFT: 2048 samples, 3 dB filter bandwidth: 18.5 Hz) on Raven Pro 1.5. A subset of three calls per male was analyzed and each measured parameter was then averaged to represent the smallest statistical unit of the analysis. Low and high frequency were measured at 20 dB (re 20 μPa) below the peak intensity, the value at which the signal energy could still be clearly distinguished from background noise in our recordings.

Synthesis of Experimental Stimuli

We synthesized stimulus calls with SoundEdit 2.0.3 software [1] and with parameters that correspond to average values of each study population. First, one "average" call was synthesized according to the average temporal and spectral parameters of each population. Second, we prepared the synthetic calls systematically changing the number of notes and frequency based on the parameters of Leticia. To test the effect of the number of notes per call and call frequency on male phonotactic response, we synthesized three series of calls with the same spectral and fine temporal properties as the first series but with different numbers of notes per call (two or three instead of four). Four-note calls consisted of average spectral and temporal parameter values of the Leticia population. To replicate each stimulus call, we randomly modified its temporal features within the population range of variation, without altering the experimental factors (call frequency and number of notes). We generated for each population between 21 and 30 replicates of the stimulus calls for each variation in the number of notes. We reanalyzed on Raven Pro 1.5 [2] all stimuli after the synthesis procedure, and these were the actual spectral and temporal values we used for the subsequent statistical analyses (for a detailed description

of this methodology see these studies [3,4]). The variation in call frequency was included to control for its effect because populations differ in the range of this parameter, and to facilitate a direct comparison with previous studies on signal recognition by receivers of *A. femoralis* and other territorial species.

Playback Experiments

During playback experiments we located a vocally active male and positioned the loudspeaker (Sony SRS-A27, Sony SRS-A47, o Sony SRS-57, Sony Corp., Tokyo) on the ground 100–230 cm away from the individual, assuming that at a larger distance that there would be excessive sound attenuation by vegetation. Playback stimuli were adjusted to a sound pressure level (SPL) between 75 and 80 dB (re 20 μPa), simulating the natural conditions of another male calling at that distance [3]. Moreover, we measured the stimulus' SPL at the focal-male position immediately after the experiment with a Sound-Level Meter (RO-1350 Roline, Rotronic AG, Bassersdorf, Switzerland). Stimulus calls were broadcasted by a battery powered loudspeaker (Sony SRS-A27, Sony SRS-A47, or Sony SRS-57, Sony Corp., Tokyo) connected to a CD player (Panasonic SI-SX320, Panasonic Corp., Secaucus, NJ, or Silva Schneider, Anif Anif, Austria CDP 286 AS). For calls of four notes, during a single experiment (lasting up to 220 s) up to 10 consecutive bouts (18–22 s per bout) were broadcasted, each bout consisting of 10 synthetic calls (9–11 s duration) followed by a silent interval (9–11 s duration).

Recognition of stimulus

Recognition of the stimulus was quantified by a phonotactic response. If the tested male crossed a perimeter of 30 cm around the loudspeaker within the 10 bouts of 10 calls of the stimulus, we stopped the experiment and declared a positive phonotactic response. The behavioral response of males was very obvious and quick. If calls were interpreted as an intruder compromising the ownership of territory, the male rapidly approached the speaker (30 cm away). When the male did not meet the response criteria and did not approach the speaker, we considered two possibilities: 1) the male did not recognize the sound as its own or 2) the male was not interested in the stimulus and/or combat. To distinguish between these two options we used a second stimulus or "control" stimulus prepared with all call parameters set to the average values. If the male approached the speaker broadcasting the second stimulus (control), we assumed that it actually failed to recognize the first stimulus as an intruder. Any subject that failed to respond to both the experimental and the control stimuli was excluded from statistical analyses. In summary, the output variable was binary. We use phonotaxis to the speaker as

the response criterion for estimating the signal recognition space because positive phonotaxis indicates three conditions. First, the individual detected signal; second, it is able to localize the stimulus; and third, the individual recognized the signal as the call of an intruder, which imposes a threat on territory ownership and therefore the owner must assume potential costs for their defense.

Bibliography

1. Weary D, Weisman R. SoundEdit v. 2.0.3. Anim Behav. 1993;45: 417–418. doi:10.1006/anbe.1993.1051
2. Bioacoustics Research Program. Raven Pro: Interactive Sound Analysis Software (Version 1.5) [Computer software] [Internet]. Ithaca, NY: The Cornell Lab of Ornithology; 2014. Available: http://www.birds.cornell.edu/raven
3. Amézquita A, Castellanos L, Hödl W. Auditory matching of male Epipedobates femoralis (Anura: Dendrobatidae) under field conditions. Anim Behav. 2005;70: 1377–1386. doi:10.1016/j.anbehav.2005.03.012
4. Amézquita A, Hödl W, Lima AP, Castellanos L, Erdtmann L, De Araújo MC. Masking interference and the evolution of the acoustic communication system in the Amazonian dendrobatid frog Allobates femoralis. Evolution (N Y). 2006;60: 1874–1887. doi:doi:10.1554/06-081.1
